# Common Single Nucleotide Polymorphisms in Clinical Cardiology and Dietary Intervention: A Narrative Review

**DOI:** 10.3390/nu18142370

**Published:** 2026-07-20

**Authors:** Jacob Michael Hands, Kevin Blain, Sahar Swidan, Leigh A. Frame

**Affiliations:** 1 Department of Pathology, University of Southern California, Los Angeles, CA 90033, USA; 2 The Office of Integrative Medicine and Health, School of Medicine and Health Sciences, The George Washington University, Washington, DC 20037, USA; kblai015@gwmail.gwu.edu (K.B.); sahar@sahar.world (S.S.); laf@drleighframe.com (L.A.F.)

**Keywords:** single nucleotide polymorphism, nutrigenetics, cardiovascular disease, lipid metabolism, APOE, FADS1/2, polygenic risk score, precision nutrition, direct-to-consumer genetic testing, dietary intervention

## Abstract

Genomic testing for rare, highly penetrant cardiovascular pathogenic mutations is well established, but its scarcity limits broader clinical utility. As cardiogenomics matures, common single nucleotide polymorphisms (SNPs) associated with cardiovascular disease (CVD) risk are increasingly accessible to clinicians and patients through both clinician-ordered and direct-to-consumer (DTC) platforms. This narrative review synthesizes evidence for six common loci—APOA1, APOE, LIPC, LPL, ANGPTL3, and FADS1/2, here termed “candidate-actionable” in the limited sense that genotype-by-diet associations have been described, but the full chain of analytical validity, clinical validity, clinical utility, and evidence-supported management has not been demonstrated—that modulate lipid metabolism and CVD risk and that show genotype-by-diet interactions in observational and small interventional studies. We frame these loci as complementary to validated polygenic risk scores (PRSs), discuss two illustrative epistatic axes (APOE ε4 × FADS major T-allele; ANGPTL3 × LPL), summarize emerging epigenetic modulators of the same loci, and propose an integrated framework combining PRS, locus-level SNPs, and epigenetic state. All locus-specific dietary considerations are explicitly framed as hypothesis-generating pending validation in adequately powered, genotype-stratified prospective trials. A comparison of consumer and clinical testing platforms—updated to reflect recent ownership and regulatory changes—is provided.

## 1. Introduction

Low-density lipoproteins (LDLs) and high-density lipoproteins (HDLs) traditionally play opposing roles in cardiovascular (CV) health; LDLs correlate with atherogenesis, while HDLs assist in reverse cholesterol transport. Very-low-density lipoproteins (VLDLs) and other triglyceride (TG)-rich particles contribute to lipid dynamics and CV risk, yet the full extent of these interactions may be difficult to characterize in isolation. Polygenic risk scores (PRSs) aggregate multiple genetic variations to predict disease likelihood, offering insight into more personalized interventions. Epigenetics—gene expression changes due to environmental and lifestyle factors—also interact with genetic predispositions to affect lipid metabolism and overall CV health, offering a modifiable risk factor that complements PRSs (developed in Section 6). These developments underscore the potential for comprehensive genetic evaluations in clinical practice.

Validated coronary artery disease (CAD) PRSs—including the Inouye metaGRS (>1.7 million variants) and the Khera genome-wide PRS—integrate many of the variants discussed in this review and provide population-level risk discrimination superior to traditional clinical scoring alone for individuals at the extremes of the PRS distribution [1,2]. However, current CAD PRSs do not provide locus-level interaction information, and they do not incorporate diet–genotype interaction effects. The locus-level review that follows is therefore complementary to, rather than redundant with, PRS-based risk assessment; it identifies actionable variants whose dietary modification cannot be inferred from a single aggregate score.

CV genomic testing for relatively rare and gross mutations impacting CVD risk is considered the standard of care and is indicated in encounters with high clinical suspicion of contributory, monogenic pathology, such as family history of structural heart disease, isolated LDL cholesterol (LDL-c) >190 mg/dL in an adult with a positive first-degree family history of atherosclerotic CV disease (ASCVD), or marked variation in plasma lipid species without apparent explanation [3]. Yet, such patients represent a limited, even if significant, number of presentations.

On the whole, it may be assumed that the routine surveillance of monogenic single nucleotide polymorphisms (SNPs) is unnecessary, costly, and without indication in the management of CVD. We argue this assumption is increasingly challenged by a growing body of low-cost genomic studies. These studies identify common, actionable genotypes implicated in individuals with high-normal mean TG and LDL-c and lower mean HDL cholesterol (HDL-c) as well as alterations in subpopulations of lipid particles, lipoproteins, particle count, particle dimensions, and particle function.

**Figure 1:**
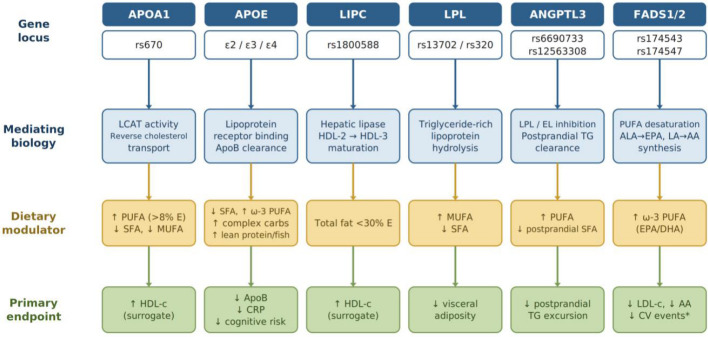
Locus → mechanism → dietary modulator → endpoint for the six common, actionable SNPs reviewed. Dietary considerations are hypothesis-generating, derived from observational and small interventional studies. Most endpoints are surrogate markers (HDL-c, LDL-c, TG); hard CV event data exist only for FADS-stratified ω-3 supplementation (* VITAL subgroup, post hoc). ALA, α-linolenic acid; LA, linoleic acid; EPA, eicosapentaenoic acid; AA, arachidonic acid; PUFA, polyunsaturated fatty acid; MUFA, monounsaturated fatty acid; SFA, saturated fatty acid; E, dietary energy. Arrows represent texpected trajectory of a particular dietary x genotype interaction.

This review targets several representative domains that involve the intersection of SNPs, dietary modification, and supplementation in applicable patients to reduce the risk associated with common genetic variants, defined here as those occurring in greater than 1% of the general population. Figure 1 provides a conceptual schematic of the locus-to-endpoint axis developed in Section 3, Section 4 and Section 5.

## 2. Scope and Methods

This narrative review does not employ a pre-registered systematic search strategy and was prepared in accordance with SANRA criteria for narrative review reporting. PubMed/MEDLINE was searched from database inception through May 2026 using combinations of gene names (APOA1, APOE, LIPC, LPL, ANGPTL3, FADS1, FADS2, PCSK9, CETP, LPA) with terms including “single nucleotide polymorphism,” “dietary intervention,” “polyunsaturated fatty acid,” “saturated fatty acid,” “lipid metabolism,” “polygenic risk score,” and “cardiovascular disease.” English-language human studies were prioritized, supplemented by backward citation tracking and expert judgment of the author team. Study selection was performed by J.M.H. with adjudication by co-authors. To constrain selection subjectivity, the following operational criteria were applied: studies were included if they (a) reported a primary association between one of the candidate loci and a lipid, lipoprotein, anthropometric, inflammatory, or cardiovascular endpoint; (b) reported either a dietary exposure/intervention or a gene–diet interaction term; and (c) were peer-reviewed, reported on humans, and were in the English language. Studies were excluded if they reported only animal or in vitro data without a corresponding human finding or if the locus association could not be linked to a dietary or lifestyle exposure. We acknowledge that, because the review was not pre-registered and the literature was not exhaustively screened against a PRISMA flow, residual selection subjectivity remains; the criteria above reduce but do not eliminate it. The six primary loci (APOA1, APOE, LIPC, LPL, ANGPTL3, FADS1/2) were selected based on three criteria: (i) common minor allele frequency (>1% in at least one major population); (ii) evidence of genotype-by-diet interaction in at least two independent cohorts or one adequately powered RCT; and (iii) biological plausibility for dietary modification through established lipid metabolism pathways. We acknowledge that criterion (ii) is met to differing degrees across loci: APOA1, APOE, LIPC, LPL, and FADS1/2 are each supported by genotype-by-diet associations in two or more independent cohorts, whereas ANGPTL3 is supported principally by a single small dietary intervention (n = 26, females only) together with mechanistic and Mendelian-randomization evidence; ANGPTL3 is therefore retained as a mechanistically motivated candidate that does not yet satisfy criterion (ii) on dietary-interaction grounds, and this limitation is flagged wherever ANGPTL3 dietary considerations are discussed. PCSK9, CETP, and LPA were included for clinical context (Section 3.7) but relegated from the primary dietary framework because they represent predominantly pharmacologic targets without established genotype-by-diet interactions amenable to non-pharmacologic intervention. Accordingly, findings represent a curated, hypothesis-generating synthesis and should not be interpreted as clinical practice guidelines. We note explicitly that the term “actionable” as throughout this review, denotes only that a genotype-by-diet association has been reported for a given locus. It does not imply that the four pillars of clinical actionability—analytical validity, clinical validity, clinical utility, and a demonstrated, evidence-supported management consequence—have been satisfied; to our knowledge, none of the loci reviewed meets all four pillars, and the term should be read as “candidate-actionable” pending prospective validation. Evidence was not formally graded using GRADE methodology; evidence-level descriptors (presented alongside each locus) reflect the authors’ qualitative assessment of study design, sample size, and replication using an adapted Oxford CEBM framework, and they have not undergone independent methodological adjudication. A study-level evidence summary for each key diet–genotype association cited is provided in Supplementary Table S2, including rsID, ancestry, sex distribution, sample size, study design, dietary exposure, primary endpoint, effect direction and magnitude, adjustment variables, replication status, and a qualitative risk-of-bias note (formal ROBINS-I or RoB 2 assessment was not performed). Qualitative descriptors used throughout the manuscript (e.g., “demonstrated,” “reported”) reflect the heterogeneity of underlying evidence rather than established causal relationships. Detailed locus-level findings arising from this search—including allele frequencies, effect estimates, and study-level evidence—are reported in Section 3 and in Supplementary Table S2 rather than in this Section 2.

## 3. Common, Actionable SNPs in Lipid Metabolism and Cardiovascular Risk

While familial hypercholesterolemia (FH) and its genetic antecedents (LDLR, PCSK9, APOB) have received significant attention, the net number of patients affected is likely less than 0.5%, though this may represent an outsized segment of the clinical population presenting with persistent hyperlipidemia, specifically elevated LDL-c [3,4]. Among the three genes chiefly implicated—LDLR, APOB, and PCSK9—LDLR remains the most common underlying variant, with an estimated heterozygous FH (heFH) disease prevalence of 1 in 250 to 1 in 303 individuals (pooled; 95% CI 1:385–1:250); LDLR variants account for >80% of genetically confirmed FH cases, but the carrier rate for LDLR sequence variants—pathogenic and benign combined—is substantially higher and should not be conflated with disease prevalence [3,4]. Genetic testing in adults with LDL-c > 190 mg/dL is indicated if there is at least one first-degree family member with a history of CVD, enabling approval for advanced lipid-lowering therapies including PCSK9 inhibitors (PCSK9i).

LDLR mutations and the diagnosis of FH generally represent, in absolute numbers, a significant clinical phenotype whose testing bears tangible implications for management, pharmaceutical therapy, and counseling. Yet, the primary agent in this form of dyslipidemia will rarely involve an FH diagnosis. Clinical dyslipidemia is primarily polygenic [5,6,7]. Putting aside recent advances in PRSs, knowledge of particular SNPs in the treatment and management of common alterations in normo-cholesterolemia is a burgeoning research area. Common variants at loci including APOA1, APOE, LIPC, LPL, ANGPTL3, and FADS1/2 represent candidates for dietary, lifestyle, and supplemental therapy (Table 1). Importantly, knowledge of such variants provides an individualized understanding of residual risk and may guide therapy. The following subsections explore the association of these variants with CVD risk factors and discuss the implications of testing with respect to dietary and non-pharmacologic treatments.

### 3.1. Apolipoprotein A-1 (APOA1)

The apparent tension between the failure of HDL-c-raising pharmacotherapy (CETP inhibitors, niacin) and the rationale for diet–genotype-informed HDL modification warrants explicit reconciliation. The failure of HDL-c-raising drugs to reduce CV events (ILLUMINATE, dal-OUTCOMES, AIM-HIGH, HPS2-THRIVE) reflects the inadequacy of HDL-c concentration as a therapeutic target when raised through mechanisms that do not improve HDL functionality [8,9,68,69]. The SNPs discussed in this and the following two sections—APOA1 rs670, LIPC rs1800588, and LPL variants—do not merely alter HDL-c concentration; they alter upstream lipid handling (reverse cholesterol transport via LCAT activity for APOA1; HDL-2/HDL-3 maturation for LIPC; TG-rich lipoprotein hydrolysis for LPL), with downstream consequences for HDL particle function, cholesterol efflux capacity, and ApoB-containing remnant burden. Dietary interventions targeted at these genotypes are therefore better conceptualized as reducing total atherogenic and metabolic burden—including reductions in VLDL remnants, postprandial TG excursion, oxidized LDL, and visceral adiposity—rather than as HDL-c-raising strategies per se. Where HDL-c is reported as the primary endpoint in the cited studies, it should be considered as a surrogate marker of altered lipid handling rather than as a direct therapeutic target; none of the cited diet–genotype studies directly measure HDL functionality (cholesterol efflux capacity, particle size, or antioxidant activity). Hard CV event data for diet–genotype interactions at these loci are absent, and the dietary considerations that follow are accordingly framed as hypothesis-generating.

APOA1 demonstrates considerable functionality, primarily modulating lecithin–cholesterol acyltransferase (LCAT) activity and, thus, altering reverse cholesterol transport. Carriers of the APOA1 rs670 minor A allele (>15–30% of carriers, according to some studies) report a greater likelihood of low HDL and, therefore, metabolic syndrome (MetS) under high monounsaturated fatty acid (MUFA) and saturated fatty acid (SFA) diets [10]. In contrast, A carriers, particularly those who are homozygous (AA), benefit significantly from the addition of polyunsaturated fatty acids (PUFAs) such that >8% PUFA intake corresponds to elevated HDL-c (reported effect magnitude: approximately 3–5 mg/dL difference in HDL-c in the Framingham cohort; clinical significance of this magnitude is uncertain and no CV event data support this finding), even relative to wild-type GG [10]. Similarly, Gomez et al. reported that A-allele carriers (APOA1–76 G > A) demonstrated reduced LDL particle size and greater susceptibility to LDL oxidation after a high-SFA diet relative to GG homozygotes, although the formal genotype × diet interaction was significant for particle size rather than for oxidation [11]. More recently, Izaola, de Luis, and Hosseini-Esfahani have added greater clarity to these observations, noting that APOA1 A-allele individuals on low-fat, hypocaloric diets prove more successful in improving HDL and markers of insulin resistance [12,70,71]. These findings suggest that dietary counseling may be modified by genetic propensities that exert independent and dependent effects, indicating the relevance of personalized nutrition research in this patient population. Importantly, the Izaola and de Luis studies employed hypocaloric diets; whether the observed improvements in HDL and insulin-resistance markers were independent of weight loss or mediated entirely by caloric restriction rather than genotype-specific fat quality has not been determined.

APOA1 rs670 is not currently included in major validated CAD PRSs (Inouye 2018; Khera 2018), reflecting its predominantly HDL-related effect; its inclusion in lipid-trait PRSs (e.g., the Global Lipids Genetics Consortium HDL-c PRS) is established [1,2].

### 3.2. Apolipoprotein E (APOE)

Though testing for the APOE ε4 variant has received significant attention as it pertains to the risk of Alzheimer’s disease (AD), there is relatively little screening for APOE variants as they relate to dietary modification. The ε4 variant is likely an ancestral remnant whose role is nuanced and may include modulating inflammatory states. While ApoE protein generally has demonstrated protective effects against lipopolysaccharide-induced lethality [13], subsequent research has shown that the ε4 isoform specifically is associated with increased sensitivity to endotoxins and increased susceptibility to sepsis progression [14,15]. This apparent paradox—wherein ApoE is broadly protective but the ε4 isoform confers heightened inflammatory risk—may reflect isoform-specific differences in receptor binding and lipopolysaccharide clearance. ApoE ε4 allele frequency varies substantially by population, ranging from approximately 6% in some East Asian populations to ~40% in certain sub-Saharan African groups, with a global average estimated near 14% [16].

With respect to CVD, ApoE is found on VLDLs, intermediate-density lipoproteins (IDLs), and chylomicrons. Particular APOE isoforms are thought to play a role in the binding affinity of these particles and their remnants to the LDL receptor-related protein (LRP) and LDL receptor (LDLr). Among the common isoforms, ApoE2 binds the LDL receptor poorly (on the order of 1–2% of E3/E4 affinity), whereas ApoE4 and ApoE3 bind with comparably high affinity; ApoE4 preferentially associates with larger, triglyceride-rich VLDL particles and is cleared through distinct kinetics, which may contribute to the relative increase in downstream VLDL remnants, such as ApoB-containing LDLs [17,18].

Dietary interventions may demonstrate varied responses by isoform. For example, ε4 carriers are acutely sensitive to the atherogenic consequences of diets high in SFAs and appear to derive outsized benefits from a relatively higher complex-carbohydrate diet and high intakes of omega-3 PUFAs and MUFAs [17,19,20]. Unprocessed red meat has been associated with null or attenuated dementia and CVD risk in several observational cohorts of ε4 carriers—including the UK Biobank (n = 493,888) [21] and a 2026 Swedish longitudinal cohort [22]—though these are observational analyses subject to confounding by dietary pattern, protein source, and socioeconomic status, and prospective RCT-level evidence is absent; clinical dietary guidance should not be modified on this basis alone [21,23,24]. This finding is coincident with the hypothesis that the ε4 allele remains an ancestral remnant whose carriers may benefit from lower-fat diets higher in low-glycemic-index carbohydrates and lean protein. A smaller but notable trial from Carvalho-Wells et al. demonstrated a potential mechanism, noting that ApoE4 carriers derived lower C-reactive protein (CRP) concentrations following a low-fat diet and favorable alterations in atherogenic lipid parameters [25]. Importantly, the benefit associated with increasing dietary protein (especially from fish), as dictated by APOE, has been reported in the Chinese Longitudinal Healthy Longevity Survey (n = 3029; mean age 77 years), in which higher dietary protein diversity and daily fish intake were associated with slower cognitive decline in ε4 carriers (OR 0.54; 95% CI 0.34–0.88, and OR 0.43 for daily fish); as an observational cognitive-aging outcome, this does not by itself establish a cardiovascular benefit [24]. Further, ε4 carriers may exhibit a significantly greater risk of cognitive decline with alcohol use [26,27,28].

Such observations are consistent with the notion that targeted genetic testing for common variants represents a feasible and powerful approach to personalized dietary modification to mitigate lipid abnormalities and reduce CVD risk. Further, randomized trials evaluating the effect of APOE disclosure on dietary modification have reported positive outcomes related to the promotion of more salutary behaviors. Therefore, personalized nutritional approaches should be coupled with genetics-informed behavioral interventions to optimize adoption in this patient population. The interaction between APOE ε4 and the FADS major T-allele—and the related question of ω-3 responsiveness—is developed in Section 3.6 and Section 4. APOE haplotypes (ε2/ε3/ε4) are typically modeled separately from genome-wide CAD PRSs given the locus’s exceptionally large effect size on LDL-c and lipoprotein clearance; whether explicit incorporation of APOE haplotype improves discrimination beyond contemporary PRSs—which already capture much of the APOE signal—remains an open question requiring prospective evaluation.

### 3.3. The Hepatic Lipase Gene (LIPC)

The LIPC gene encodes hepatic lipase, likely primarily responsible for the hydrolysis of TG-rich lipoproteins, including HDL. The LIPC gene has been implicated in coronary artery disease (CAD), with underactivity associated with enhanced risk even in the context of moderately increased average HDL-c [29,30,31]. LIPC is thought to mediate HDL maturation and increase the conversion of HDL-2 to TG-depleted HDL-3 particles; thereby, its dysregulation is thought to significantly increase CAD risk [31]. Minor LIPC allele frequencies are estimated to be >20%, depending on ethnicity [30,32,33].

Dietary fat is a known promoter of HDL-c [34,35]. Yet, in this subgroup of minor allele carriers (rs1800588), the addition of dietary fat may, counterintuitively, have unfavorable consequences with respect to HDL particle number and function. As Ordovas et al. have demonstrated, TT minor-allele carriers are best advised to consider total fat consumption below 30% to optimize HDL-c, while C/T and CC carriers reported no significant alteration associated with SFA consumption [36]. Caution is warranted: the Ordovas Framingham cohort is predominantly European-American; these findings may not generalize across ancestries. In general, increases in fat intake in this population coincide with lower levels of HDL-c, producing an unfavorable profile. This finding has been replicated by Smith et al. and remains a valid clinical consideration and an area ripe for additional research [37]. The macronutrient chosen to replace dietary fat is clinically relevant; substitution with refined carbohydrates could produce neutral or adverse effects on atherogenic dyslipidemia, and the cited studies do not specify replacement macronutrient composition. Both key studies cite HDL-c as the primary endpoint; hard CV event data in LIPC variant carriers are absent. LIPC rs1800588 is included as a contributing variant in the Global Lipids Genetics Consortium HDL-c PRS; it is not independently weighted in current CAD PRSs.

### 3.4. Lipoprotein Lipase (LPL)

Lipoprotein lipase (LPL) is an enzyme with significant expression in cardiac and skeletal muscle. LPL functions to hydrolyze TG-replete lipoproteins such as chylomicrons and VLDLs. While absent LPL activity (Type 1 familial chylomicronemia syndrome) is a rare mutation characterized by markedly elevated atherogenic burden, several common LPL variants exist in the general population, with prevalence estimated to be 15–45%, though likely with significant heterogeneity [38,39,40].

Considerations differ for those with the minor allele, including reduced SFA intake. In a small cohort sample, Hammad et al. documented a significant association between common LPL variant rs13702 and varied response to SFA reduction and MUFA consumption, with a diet high in MUFAs associated with significant reductions in visceral adiposity among major-allele CC carriers, whereas those carriers consuming diets high in SFAs demonstrated increased visceral adiposity [41]. Hannon et al., in a small pilot study (n = 101; insufficient power for subgroup analyses) of healthy adults, added credence to this finding, documenting significant increases in HDL concentrations only in AG/GG risk-allele carriers with high fat intake [42]. In a larger cohort of more than 1000 participants, Ma et al. explored the effect of another common LPL variant, rs320, and its interaction with high-PUFA diets, documenting significant reductions in body mass index (BMI) and waist circumference only in major-allele (GG) female carriers [43]. The sex-specific restriction in the Ma et al. finding limits generalizability to male patients.

As fat intake increases LPL activity, understanding of personal SNPs pertaining to LPL activity may help elucidate the sometimes contradictory results between elevated HDL-c concentrations and fat intake in diverse populations. Notably, LPL expression in skeletal muscle, cardiac tissue, and adipose tissue has different metabolic consequences; the tissue compartment in which the SNP effect dominates is not addressed by the dietary intervention studies cited. LPL locus variants contribute to TG-trait PRSs and to the Inouye metaGRS for CAD [1].

### 3.5. Angiopoietin-like Protein 3 (ANGPTL3)

An important drug target for treating dyslipidemia is ANGPTL3 (angiopoietin-like protein 3), owing to its key role in regulating lipid metabolism by inhibiting LPL and endothelial lipase (EL). These enzymes are vital for the hydrolysis and clearance of triglycerides, LDL-c, and HDL-c, thereby impacting circulating lipid concentrations [44]. Moreover, there are SNPs that reduce the activity of ANGPTL3, leading to enhanced LPL function and ultimately resulting in lower serum lipids. For instance, individuals with loss-of-function (LOF) mutations in the ANGPTL3 gene present reduced postprandial triglyceride responses after consuming high-fat meals [45]. Conversely, specific mutations (rs6690733 and rs12563308) in ANGPTL3 promoter regions are thought to increase promoter activity and consequently increase ANGPTL3 expression [44]; however, functional validation data (e.g., luciferase reporter assays, allele-specific expression in human tissues) have not been reported for these specific variants, and the risk assignment is based on association rather than a demonstrated molecular mechanism.

A randomized controlled trial evaluating the impact of a 7-day PUFA-rich diet on biomarkers of lipid metabolism in healthy female participants found that adherence to a high-PUFA diet was associated with lower ANGPTL3 and ANGPTL8 levels [46]. This study enrolled 26 participants total (16 PUFA, 10 control) and was conducted exclusively in healthy females; results cannot be generalized to males or to clinical populations with dyslipidemia. ANGPTL8 is known to decrease adipose triglyceride lipase (ATGL), a crucial enzyme in lipolysis (the breakdown of triacylglycerols via hydrolysis) in adipocytes. This indicates that dietary PUFA intake may regulate lipid metabolism by affecting ANGPTL-mediated ATGL activity in adipose tissue. The sex-specific results (ANGPTL3 and ANGPTL8 reductions were observed only in females, not males, within the same trial) underscore the influence of sex hormones on PUFA metabolism pathways; dietary PUFA recommendations based on this single small trial should not be extrapolated to male patients or clinical populations pending larger, sex-stratified RCTs. Notably, this trial was not designed to test SNP-specific interactions; the relevance of these findings to carriers of ANGPTL3 gain-of-function promoter variants specifically is extrapolated from the general PUFA–ANGPTL3 relationship and has not been directly demonstrated. ANGPTL3 variants are currently underrepresented in CAD PRSs given the rarity and variable annotation of the variants; loss-of-function carriers are typically identified through targeted sequencing rather than PRS.

### 3.6. Fatty Acid Desaturases (FADS1/2)

The fatty acid desaturases (FADS1/2) mediate the conversion of several steps in PUFA synthesis. Variants in FADS and how they pertain to health outcomes—including CVD risk, diabetes, and cancer—are among the most well-described polymorphisms in the general population, with large cohorts (n > 100,000) demonstrating the significant effect of this locus on PUFA synthesis and, therefore, synthetic PUFA activity [47,48,49].

The FADS major allele is an ancestral variant whose conversion of plant alpha-linolenic acid (ALA) and linoleic acid (LA) into eicosapentaenoic acid (EPA) and arachidonic acid (AA), respectively, represented a necessary biosynthetic pathway to derive these essential PUFAs. In the course of migration, it is thought that selection pressures which once preserved the major FADS locus were no longer present, resulting in a minor-derived allele with greater frequency outside the African continent [49]. In large cohorts, the diminished capacity of FADS1/2 minor alleles has appeared protective in multiple phenotypes, particularly CVD [50,51]. These carriers boast significantly lower atherogenic lipoproteins, including LDL, and inflammatory species, including AA [52,53,54,55,56,57,58,59,60,61,62,63]. The particular FADS1/2 variant may explain upwards of 18.6% of the total variation in plasma AA [63]. Of note, there is inconsistency reported with FADS1/2 carriers and LDL plasma concentrations. It is thought that discrepancies in LDL-c among minor-allele carriers are paradoxically explained by higher omega-3 consumption in this cohort [64,65]. Throughout, we refer to the FADS1 index SNP rs174547 (T > C); rs174543 and rs174537 are tightly linked proxies within the same haplotype block and are reported here under the common rs174547 reference for consistency of allele direction. In the Malmö Diet and Cancer Study cohort (4635 participants), the minor C allele (rs174547) was associated with significantly lower LDL-c, an effect which was attenuated (i.e., the LDL-c difference between minor and major allele carriers was narrowed) by higher ambient omega-3 intake; no dose–response relationship was reported [66].

Conversely, major T-allele carriers (rs174547) have shown significantly greater risk for adverse CV outcomes and derive significantly greater benefit from increasing omega-3 consumption. As Chilton et al. note, large studies of omega-3 may be confounded by varied responses due to FADS major-allele carriers deriving outsized benefit from diets higher in omega-3, explaining improved outcomes in particular subgroups [67]. The VITAL Study—a large, null trial at the primary cardiovascular prevention level (Manson et al., 2019; N = 25,871; overall HR for major CVD events not significant) [72]—demonstrated a markedly greater reduction in myocardial infarction risk (approximately 77%) in the African American subgroup, a population with a higher prevalence of FADS major-allele carriers, consistent with the hypothesis that ancestry-linked FADS genotype modulates ω-3 responsiveness [67]. This subgroup effect, mechanistically attributed by Chilton et al. to elevated FADS major T-allele prevalence in individuals of African ancestry, remains a plausible but unconfirmed hypothesis pending prospective genotype-stratified trials. However, in a study of 1464 CVD-free participants at baseline, Bäck et al. reported that pulse-wave velocity—a marker of vascular health—was improved in major T-allele carriers (rs174547) with increasing omega-3 PUFA intake [65].

The implications of these findings are that FADS alleles may have outsized implications for BMI, lipid abnormalities, and other CVD risk factors. Thus, dietary advisement may be sharply contingent on knowledge of these variants, supporting personalized nutrition research. FADS1/2 variants are included in the GLGC LDL-c and TG PRSs and in metaGRS-style CAD PRSs; the FADS locus is among the most strongly weighted loci in PUFA-trait PRSs.

### 3.7. Other Notable Genetic Variants: PCSK9, CETP, and LPA

Several genetic variants, such as PCSK9, CETP, and LPA, are gaining attention due to their association with CV outcomes and treatment responses. Cohen et al. (2006) [73] demonstrated in the Atherosclerosis Risk in Communities (ARIC) cohort that PCSK9 nonsense mutations—carried by 2.6% of Black participants—were associated with a 28% reduction in mean LDL-c and an 88% reduction in coronary heart disease risk (HR 0.11; 95% CI 0.02–0.81) over 15 years; a distinct PCSK9 variant in white participants (3.2% prevalence) was associated with a 15% LDL-c reduction and a 47% CHD risk reduction (HR 0.50; 95% CI 0.32–0.79). These are observational findings from a biracial cohort and cannot be equated in magnitude to randomized trial evidence [73]. The landmark FOURIER trial subsequently demonstrated that therapeutic PCSK9 inhibition with evolocumab reduced major adverse cardiovascular events by 15% in patients with established atherosclerotic disease on statin therapy, providing the prospective RCT-level confirmation that the observational ARIC findings could not [74]. Additionally, the GLAGOV trial showed that PCSK9 variants influenced plaque regression in response to evolocumab therapy [75]. These findings reinforce the clinical relevance of these variants and genetics-informed counseling in CV management. These loci are included for clinical context but are not integrated into the dietary recommendations in Table 2, as they represent primarily pharmacologic—rather than dietary—targets.

## 4. Epistasis and the Polygenic Context

**Figure 2:**
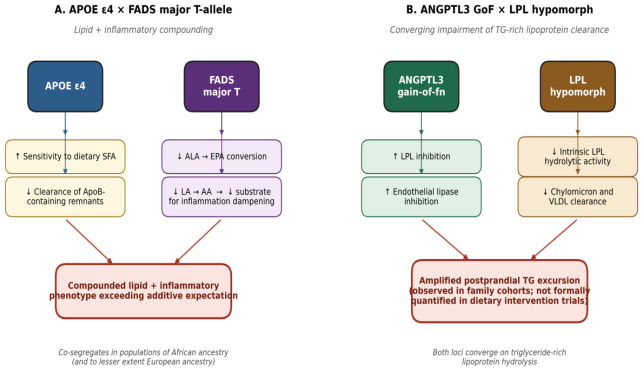
Two epistatic axes among the loci reviewed. (**A**) APOE ε4 × FADS major T-allele: compounded lipid and inflammatory phenotype exceeding additive expectation. (**B**) ANGPTL3 gain-of-function × LPL hypomorph: converging impairment of triglyceride-rich lipoprotein clearance amplifies postprandial TG excursion. Neither axis is currently captured as an interaction term in validated CAD polygenic risk scores. Arrow represents expected dietary effect.

The SNPs reviewed in Section 3 are presented individually for pedagogical clarity, but their clinical relevance is shaped by interactions that the single-variant frame obscures (Figure 2). APOE ε4 and the FADS major T-allele co-segregate in populations of African and, to a lesser extent, European ancestry, and their co-occurrence is associated with a compounded lipid and inflammatory phenotype. No formal statistical test for interaction (multiplicative or additive scale) has been reported for this axis in any genotype-stratified dietary trial; the described compounding remains a mechanistic hypothesis, not an empirically validated interaction. Mechanistically, ε4 confers heightened sensitivity to dietary saturated fat and reduced clearance of ApoB-containing remnants, while the FADS major T-allele limits endogenous conversion of plant-derived α-linolenic acid (ALA) and linoleic acid (LA) to eicosapentaenoic acid (EPA) and arachidonic acid (AA), potentially narrowing the substrate available for synthesis of anti-inflammatory long-chain PUFA derivatives. We emphasize that this mechanistic sequence is inferred from the separate established functions of each variant; the proposed convergence has not been directly demonstrated in carriers of both variants, and no direct molecular or interventional evidence establishes the compounded inflammatory phenotype described. The convergence of ANGPTL3 and LPL on triglyceride-rich lipoprotein clearance creates a related epistatic axis; ANGPTL3 gain-of-function variants and LPL hypomorphic variants individually impair TG hydrolysis, and their co-occurrence amplifies postprandial TG excursion in a manner that has been observed in family-based cohorts but has not been formally quantified in dietary intervention trials. These interactions are partially captured by validated CAD polygenic risk scores—most notably the Inouye metaGRS (2018), which incorporates >1.7 million variants including those reviewed here, and the Khera genome-wide PRS (2018)—but neither PRS currently provides interaction-aware risk stratification at the individual-locus level [1,2]. Clinical translation of the dietary considerations discussed therefore requires acknowledgment that the patient before the clinician is a composite of interacting variants, and that single-SNP dietary advisement is a simplification of the underlying biology.

## 5. Epigenetic Modulation of the Reviewed Loci

Beyond inherited sequence variation, gene expression at several of the loci reviewed is modulated by epigenetic mechanisms responsive to dietary and environmental exposures. The FADS locus undergoes differential DNA methylation in response to dietary PUFA intake, with methylation patterns at CpG sites within FADS2 modifying the phenotypic expression of the rs174547 genotype on plasma fatty acid composition [76]. APOE promoter methylation has been associated with both Alzheimer’s disease risk and lipid phenotype expression in ε4 carriers, reported as an association only [77]. We caution that no direct evidence demonstrates that altering APOE promoter methylation through dietary or lifestyle exposure modifies CV or neurocognitive risk in ε4 carriers; the inference that this risk is “partially modifiable” through methylation-altering exposures is speculative and is not supported by interventional data. Methylation at the LPL locus has been linked to obesity-related metabolic dysfunction and may explain inter-individual variation in postprandial TG response not accounted for by genotype alone [78]. These findings reinforce the rationale for sustained dietary and lifestyle intervention even in carriers of risk alleles; the inherited genotype is fixed, but the methylation state—and thus the realized phenotype—remains responsive to modifiable exposures. Importantly, none of the cited studies demonstrate that diet-induced methylation changes at these specific loci causally alter CVD risk; correlation between methylation state and phenotype does not establish that methylation is a clinically actionable modifiable target. No validated clinical assays exist for FADS2, APOE, or LPL methylation with established reference ranges, test–retest reliability, or proven utility for guiding dietary changes. Furthermore, the cited LPL methylation study did not measure methylation in the same individuals with known LPL genotypes, so the unique contribution of methylation independent of genotype remains unknown. Integration of epigenetic data with SNP genotype and PRS represents an emerging research direction for cardiovascular risk refinement, developed in Section 6.

## 6. Integrated Framework: PRS, Locus-Level SNPs, and Epigenetics

**Figure 3:**
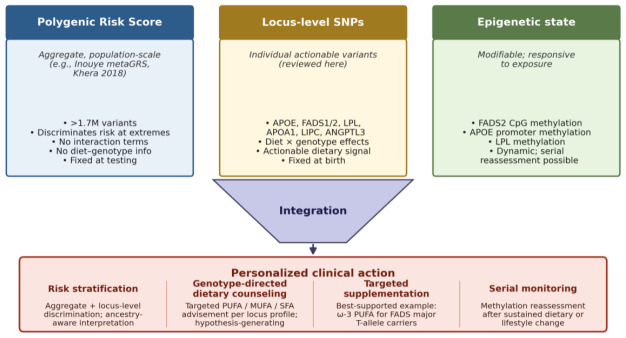
Integrated framework for cardiovascular risk refinement. Three complementary inputs—polygenic risk score, locus-level SNPs, and epigenetic state—converge on personalized clinical action. PRS, polygenic risk score; CpG, cytosine-guanine dinucleotide. All clinical actions are framed as adjuncts to standard guideline-directed cardiovascular care and require independent verification in CLIA/CAP-certified laboratories before clinical decision-making.

Three complementary streams of genomic information are now accessible to clinicians and patients (Figure 3). Validated polygenic risk scores provide aggregate, population-scale discrimination but no diet–genotype interaction information. Locus-level SNPs—the focus of this review—provide actionable, individualized signals where genotype-by-diet interactions have been described. Epigenetic state, particularly DNA methylation at FADS2, APOE, and LPL CpG sites, adds a dynamic, modifiable layer responsive to sustained exposure. Integration of these three streams supports a more nuanced clinical workflow than any single stream alone: PRS for risk stratification at the population extremes; locus-level SNPs for genotype-directed dietary counseling and targeted supplementation (e.g., ω-3 PUFA in FADS major T-allele carriers, the best-supported example in this review); and methylation reassessment as an experimental research tool for monitoring response to intervention (no validated clinical methylation assays currently exist for this purpose). All such integration is currently a conceptual research framework requiring validation of discrimination, calibration, reclassification, decision-curve benefit, ancestry transferability, and clinical thresholds before adoption into clinical practice; it remains adjunctive to standard guideline-directed cardiovascular care.

## 7. Practical Considerations for Genetic Testing

### 7.1. Sex-Specific Effects on Lipid Phenotype

Several studies reviewed here reported associations that differed by sex—for example, the LPL rs320 adiposity association (observed in females only) and sex-stratified APOA1 and ANGPTL3 findings. The mechanisms underlying these reported differences are not established; while sex-hormone effects on gene expression and lipid metabolism are biologically plausible, the cited studies did not test this mechanism, and several sex-specific findings were derived from underpowered subgroups without formal sex-by-genotype interaction testing. These observations should therefore be regarded as hypothesis-generating signals that motivate prospectively sex-stratified trials rather than as a basis for sex-specific dietary or pharmacological prescription at present.

### 7.2. Cost-Effectiveness, Accessibility, and Platform Selection

Cost-effectiveness and accessibility are vital considerations when implementing genetic testing for CV risk assessment. Clinicians may benefit from guidelines on when genetic tests are warranted, with recommendations for reliable testing platforms that perform appropriate allele assessments. This guidance would aid in resource allocation and ensure that genetic testing is used effectively, supporting both patient care and clinical decision-making.

Clinical application of the SNPs reviewed requires testing performed in laboratories certified under the Clinical Laboratory Improvement Amendments (CLIA) and ideally accredited by the College of American Pathologists (CAP). Direct-to-consumer (DTC) genotyping platforms provide informational results that should not guide clinical decision-making without independent verification in a CLIA/CAP-certified laboratory. Clinician-ordered panels designed for monogenic cardiovascular conditions (e.g., familial hypercholesterolemia, cardiomyopathies, channelopathies) capture some but not all of the common polygenic variants discussed in this review; SNP-array or whole-genome platforms are required for comprehensive assessment of the loci reviewed here. A comparison of available platforms—covering DTC, hybrid, and clinician-ordered models and updated to reflect recent ownership and regulatory changes including the 23 March 2025 and Me Chapter 11 filing and subsequent acquisition by TTAM Research Institute—is provided in Supplementary Table S1. Platform information is current as of May 2026; availability, ownership, regulatory status, and product offerings may change rapidly and should be independently verified by clinicians before use. Inclusion of any platform does not constitute endorsement by the authors.

## 8. Future Research Directions and Limitations

Further research on this topic should focus on advancing genomic databases and enhancing predictive models to assess CVD risk. The adoption of these genetic screenings into everyday clinical practice is contingent upon both the accessibility and affordability of these new screening procedures. This raises questions about the feasibility of implementing these new tools, as widespread adoption typically requires evidence of direct causation rather than mere correlation.

Additional priorities for future research, framed as testable hypotheses for specific patient populations (primary prevention with intermediate ASCVD risk, unexplained dyslipidemia, individuals at PRS extremes, or patients presenting with DTC genetic results), include: (1) Hypothesis: APOE ε4 carriers show greater ApoB/LDL-c response to saturated-fat reduction than non-carriers in prospective RCTs powered for hard CV endpoints (myocardial infarction, stroke, CVD mortality); (2) Hypothesis: FADS1/2 major-allele carriers show differential lipid and inflammatory response to ω-3 supplementation in genotype-stratified trials; (3) Hypothesis: ANGPTL3 and LPL risk-variant carriers show sex-specific differences in postprandial triglyceride response to dietary fat composition, testable in sex-stratified intervention trials; (4) Hypothesis: co-occurrence of APOE ε4, FADS1/2 major-allele, and LPL risk variants produces a cumulative lipid phenotype exceeding additive single-locus expectations, testable through formal gene–gene interaction analysis in adequately powered cohorts; (5) Hypothesis: adding the locus-level SNPs reviewed to a validated CAD polygenic risk score improves reclassification and decision-curve benefit beyond the PRS alone; (6) Hypothesis: gut-microbiome composition modifies the association between FADS and APOE genotypes and circulating PUFA levels, testable in genotype-stratified microbiome studies; and (7) Hypothesis: the magnitude of genotype-by-diet interaction at each locus is large enough relative to the main effects of genotype alone and diet alone to be clinically meaningful—a quantitative comparison that, to our knowledge, has not been formally reported and is a prerequisite for establishing clinical importance.

Several limitations of the present review warrant explicit acknowledgment. First, this is a narrative synthesis not governed by a pre-registered systematic search; selection of evidence reflects authors’ judgment. Second, evidence-level descriptors are an adapted CEBM qualitative assessment and not formally graded under GRADE. Third, most dietary considerations rest on observational data or small interventional trials with surrogate endpoints; hard CV event data stratified by the loci reviewed are essentially absent outside post hoc subgroup analyses. Fourth, ancestry representation is uneven across the underlying literature; European-American cohorts predominate, and generalizability across ancestries is therefore limited. Fifth, sex-specific effects are reported in several studies but are inconsistently powered. Sixth, most cited observational studies examined multiple SNPs, dietary components, and lipid endpoints without correction for false discovery; the risk of type I errors in the reported genotype-by-diet interactions is substantial and unquantified. Seventh, no functional studies (e.g., knockout models, human primary cell assays, iPSC-derived hepatocytes) directly demonstrating how the specific SNPs alter transcription factor binding, mRNA stability, or protein activity are cited for APOA1 rs670, LIPC rs1800588, or LPL rs13702; the associations reviewed rest on population-level statistical associations. Eighth, UK Biobank analyses may be subject to collider bias from conditioning on survival to enrollment age and availability of genetic data. These limitations underscore that the dietary considerations summarized in Table 2 are hypothesis-generating and should not be interpreted as clinical practice guidelines.

## 9. Conclusions

As low-cost genetic testing becomes standard, the understanding of personal genomics and modifiable SNPs will become increasingly important. In this review, we identified and described several common—yet scarcely considered in clinical practice—genetic variants that are associated with increased CVD risk and for which genotype-by-diet interactions have been described in observational and small interventional studies. These variants represent biologically plausible candidates for future genotype-stratified dietary intervention research (e.g., ω-3 fatty acid supplementation for FADS1/2 major T-allele carriers, as the best-supported example in this review), though current evidence does not support routine genotype-directed dietary prescription for these loci outside research settings or carefully contextualized clinical counseling.

The correlation between these variants and mortality is unclear and sometimes counterintuitive. Further research should focus on large-scale longitudinal studies to validate SNP-based dietary considerations and assess their real-world impact on CVD prevention. Additionally, investigating the interaction between these genetic variants and gut microbiome composition may provide novel insights into lipid metabolism and cardiovascular health. Moving toward a more personalized approach to medicine—i.e., precision medicine—may reduce CVD burden and improve health outcomes in part by integrating comprehensive genomic screening into routine practice, which requires addressing challenges related to cost, accessibility, and clinical utility. Developing standardized guidelines for interpreting and applying genomic data in CV care will be crucial for widespread adoption. All dietary considerations arising from this review should be regarded as hypothesis-generating pending prospective validation in adequately powered, genotype-stratified clinical trials.

## Figures and Tables

**Table 1:** Summary of actionable SNPs reviewed, estimated minor allele frequencies, and key functional associations.

Evidence Level (Oxford CEBM, Adapted)	Primary Functional Association	Refs.	MAF Range	Key SNP(s)	Gene
Level 3—Cohort and small RCT data; replicated in Framingham subset; surrogate endpoint (HDL-c) only.	Modulates LCAT; affects HDL-c response to MUFA/SFA vs. PUFA diets	[8,9,10,11,12]	15–30%	rs670	*APOA1*
Level 2 (lipid/AD outcomes); Level 3 (dietary interaction). UK Biobank, SNAC-K; ω-3 × ε4 RCT data limited.	Lipoprotein receptor binding affinity; dietary fat sensitivity	[13,14,15,16,17,18,19,20,21,22,23,24,25,26,27,28]	ε4 ~6% (East Asian) to ~40% (sub-Saharan African); global ~14%	ε2/ε3/ε4	*APOE*
Level 3—Framingham + Smith replication; European-American only; HDL surrogate.	Hepatic lipase activity; HDL maturation and CAD risk	[29,30,31,32,33,34,35,36,37]	>20%	rs1800588	*LIPC*
Level 4—Small pilots (Hannon *n* = 101); inconsistent sex effects; replication limited.	TG-rich lipoprotein hydrolysis; visceral adiposity	[38,39,40,41,42,43]	15–45%	rs13702, rs320	*LPL*
Level 4—Single 7-day RCT, *n* = 26, females only; not generalizable.	LPL/EL inhibition; postprandial TG clearance	[44,45,46]	Variable	rs6690733, rs12563308	*ANGPTL3*
Level 2 (mechanism); Level 3 (clinical outcome). Large GWAS/MR (*n* >100,000); VITAL subgroup post hoc.	PUFA desaturation; AA/EPA synthesis; LDL-c and inflammation	[47,48,49,50,51,52,53,54,55,56,57,58,59,60,61,62,63,64,65,66,67]	~30–60%	rs174547 (proxies: rs174543, rs174537)	*FADS1/2*

Evidence levels adapted from Oxford CEBM Levels of Evidence (2011) for therapy/prevention questions. Level 1: systematic review of RCTs; Level 2: individual RCT or large observational with dramatic effect; Level 3: non-randomized cohort or follow-up study; Level 4: case series or small pilot; Level 5: mechanism-based reasoning. Levels reflect authors’ qualitative assessment and have not undergone formal independent methodological adjudication. MAF, minor allele frequency; LCAT, lecithin–cholesterol acyltransferase; MUFA, monounsaturated fatty acid; SFA, saturated fatty acid; PUFA, polyunsaturated fatty acid; HDL-c, high-density lipoprotein cholesterol; CAD, coronary artery disease; TG, triglyceride; LPL, lipoprotein lipase; EL, endothelial lipase; AA, arachidonic acid; EPA, eicosapentaenoic acid; LDL-c, low-density lipoprotein cholesterol; AD, Alzheimer’s disease; MR, Mendelian randomization.

**Table 2:** Preliminary genotype-specific dietary considerations based on available observational and interventional evidence (hypothesis-generating; not for direct clinical application without independent prospective validation).

Dietary Factors Associated with Less Favorable Profile	Dietary Consideration (Hypothesis-Generating)	Risk Genotype	Gene
High-SFA and high-MUFA diets associated with lower HDL	Higher PUFA intake (>8% of energy) associated with elevated HDL-c; low-fat, hypocaloric diets may improve insulin-resistance markers [effect size modest: ~3–5 mg/dL ΔHDL-c; no CV event data]	rs670 A allele	*APOA1*
Diets high in SFA; excessive alcohol	Higher complex carbohydrate, ω-3 PUFA, MUFA, and fish protein intake associated with favorable profile	ε4 carriers	*APOE*
High-fat diets (>30% energy) associated with lower HDL	Total fat < 30% of energy associated with higher HDL-c [Framingham cohort; European-American ancestry only; HDL surrogate only]	rs1800588 TT	*LIPC*
High-SFA diets (associated with increased visceral adiposity)	Studies suggest MUFA-favored diets associated with reduced visceral adiposity [small pilot studies; insufficient power; sex-specific effects unresolved]	rs13702 minor allele	*LPL*
High-SFA postprandial loading	High-PUFA diet may reduce ANGPTL3/8 expression [n = 26 females only; 7-day RCT; not generalizable to males]	Promoter gain-of-function variants	*ANGPTL3*
Low ω-3 intake (associated with attenuation of protective effect)	Higher ω-3 PUFA intake associated with reduced CV risk [supported by large GWAS and VITAL subgroup; MR evidence consistent]	Major T allele (rs174547)	*FADS1/2*

PUFA, polyunsaturated fatty acid; SFA, saturated fatty acid; MUFA, monounsaturated fatty acid; HDL-c, high-density lipoprotein cholesterol; GWAS, genome-wide association study; MR, Mendelian randomization. Italicized annotations indicate evidence quality and key limitations.

## Data Availability

No new data were created or analyzed in this study. Data sharing is not applicable to this article.

## Supplementary Materials

The following supporting information can be downloaded at: https://www.mdpi.com/article/10.3390/nu18142370/s1, Table S1: Comparison of Consumer and Clinical Genetic Testing Platforms Relevant to CVD-Associated SNP Assessment; Table S2: Study-level evidence summary for the key diet–genotype associations cited in this review. All values are taken directly from the cited primary publications (verified against PubMed records); “NR” denotes a parameter not reported in the source. Sample sizes are the number of participants analyzed for the genotype-stratified comparison. Many primary studies did not report a formal interaction coefficient with confidence interval or a correction for multiple comparisons; these gaps are shown as “NR” rather than imputed, and are themselves relevant to the strength of the evidence. No formal ROBINS-I or RoB 2 instrument was applied; risk-of-bias entries are the authors’ structured qualitative judgment. Genome build and strand are not listed per row because the cited studies report variants by rsID only; readers should resolve each rsID against dbSNP (current build) before clinical interpretation.

## Abbreviations

AA, arachidonic acid; AD, Alzheimer’s disease; ALA, α-linolenic acid; ApoB, apolipoprotein B; ASCVD, atherosclerotic cardiovascular disease; ATGL, adipose triglyceride lipase; BMI, body mass index; CAD, coronary artery disease; CAP, College of American Pathologists; CEBM, Centre for Evidence-Based Medicine; CETP, cholesteryl ester transfer protein; CLIA, Clinical Laboratory Improvement Amendments; CRP, C-reactive protein; CV, cardiovascular; CVD, cardiovascular disease; DTC, direct-to-consumer; EL, endothelial lipase; EPA, eicosapentaenoic acid; FH, familial hypercholesterolemia; GLGC, Global Lipids Genetics Consortium; HDL, high-density lipoprotein; HDL-c, high-density lipoprotein cholesterol; IDL, intermediate-density lipoprotein; LA, linoleic acid; LCAT, lecithin–cholesterol acyltransferase; LDL, low-density lipoprotein; LDL-c, low-density lipoprotein cholesterol; LDLr, LDL receptor; LRP, LDL receptor-related protein; LPL, lipoprotein lipase; MAF, minor allele frequency; MR, Mendelian randomization; MUFA, monounsaturated fatty acid; NGS, next-generation sequencing; PCSK9, proprotein convertase subtilisin/kexin type 9; PRS, polygenic risk score; PUFA, polyunsaturated fatty acid; RCT, reverse cholesterol transport (Section 3.1)/randomized controlled trial (elsewhere); SFA, saturated fatty acid; SNP, single nucleotide polymorphism; TG, triglyceride; VLDL, very-low-density lipoprotein; WES, whole-exome sequencing; WGS, whole-genome sequencing.
