# Supplementary material for: Common Single Nucleotide Polymorphisms in Clinical Cardiology and Dietary Intervention: A Narrative Review
Source: Nutrients. 2026 Jul 20;18(14):2370. doi: 10.3390/nu18142370 (PMC13415203; doi:10.3390/nu18142370)
Supplement: Supplementary file 1 [file nutrients-18-02370-s001.zip › supplementary Table S1.pdf]

Supplementary Material

Supplementary Table S1. Comparison of Consumer and Clinical Genetic Testing Platforms Relevant to CVD-Associated SNP Assessment

Note on currency: *Commercial information in this table was verified as of May 2026 and is subject to rapid change. Platform availability, ownership, regulatory status, and product offerings may have changed since publication. Clinicians and patients should verify current status independently before relying on platform-specific information.*

Platform	Model	Technology	CLIA/CAP	CV panels	Genetic counseling	Key considerations
23andMe (TTAM Research Institute)	DTC	SNP array (~600K)	FDA-authorized	Limited	No	Filed Chapter 11 bankruptcy March 2025; assets acquired by TTAM Research Institute; data stewardship uncertain.
Nebula Genomics	DTC	WGS (30×)	CLIA	350+ reports	No	Informational only; not clinician-ordered; subscription model; requires independent clinical interpretation.
SelfDecode	DTC (hybrid)	SNP array + advanced imputation (~200M variants); some Quest-administered labs	CLIA (via Quest)	PRS-based reports for APOE, FADS, LPL, CETP, PCSK9, LIPC, ANGPTL3, LPA	No (informational; AI recommendations)	Polygenic-risk-score reports available; ancestry-aware risk scoring; subscription + bundle pricing; HIPAA/GDPR compliant; explicitly does not sell user data.

Platform	Model	Technology	CLIA/CAP	CV panels	Genetic counseling	Key considerations
						Informational only; not for clinical decision-making without CLIA/CAP verification.
Invitae (Labcorp)	Clinician	NGS panels	CLIA/CAP	Yes	Yes	Comprehensive cardiology panels; physician portal; 10–21 day TAT; primarily monogenic—not optimized for polygenic SNP screening.
Ambry Genetics	Clinician	SNP array, NGS, WES	CLIA/CAP	Yes	Yes	Results to ordering physician only; AmbryShare data sharing; clinician ordering required.
Color Health	Hybrid	Targeted NGS	CLIA	Yes	Yes	NIH "All of Us" partner; low-cost; auto-shares with provider; clinician-shared results;

Platform	Model	Technology	CLIA/CAP	CV panels	Genetic counseling	Key considerations
						covers common SNP loci discussed in this review.
GeneDx	Clinician	WGS / WES	CLIA/CAP	Limited	Yes	Rare disease focus; FDA Breakthrough Device (Oct 2025); AI variant interpretation (Multiscore); WGS captures common SNPs but not primary use case.
Myriad Genetics	Clinician	NGS panels	CLIA/CAP	Hereditary	Yes	myRisk (cancer/CV); GeneSight (PGx); strong insurance coverage; CV focus primarily hereditary cancer—not polygenic lipid SNPs.
Natera	Clinician	cfDNA, SNP array	CLIA	Emerging	Yes	Primarily prenatal/oncology; expanding into cardiogenomics;

Platform	Model	Technology	CLIA/CAP	CV panels	Genetic counseling	Key considerations
						limited CV SNP coverage for SNPs reviewed here.

DTC, direct-to-consumer; WGS, whole-genome sequencing; NGS, next-generation sequencing; WES, whole-exome sequencing; CLIA, Clinical Laboratory Improvement Amendments; CAP, College of American Pathologists; TAT, turnaround time; PRS, polygenic risk score; PGx, pharmacogenomics; CV, cardiovascular. Inclusion of any platform does not constitute endorsement by the authors or the journal. For research-grade reproducibility of locus-level results, independent verification in a CLIA/CAP-certified laboratory remains the standard. Commercial information is subject to rapid change; verify current status independently before clinical use. No author has received financial support, consulting fees, or equity from any genetic testing platform named in this table.
