# Supplementary material for: Common Single Nucleotide Polymorphisms in Clinical Cardiology and Dietary Intervention: A Narrative Review
Source: Nutrients. 2026 Jul 20;18(14):2370. doi: 10.3390/nu18142370 (PMC13415203; doi:10.3390/nu18142370)
Supplement: Supplementary file 1 [file nutrients-18-02370-s001.zip › supplementary Table S2.pdf]

Supplementary Table S2. Study-level evidence summary for the key diet–genotype associations cited in this review. All values are taken directly from the cited primary publications (verified against PubMed records); "NR" denotes a parameter not reported in the source. Sample sizes are the number of participants analyzed for the genotype-stratified comparison. Many primary studies did not report a formal interaction coefficient with confidence interval or a correction for multiple comparisons; these gaps are shown as "NR" rather than imputed, and are themselves relevant to the strength of the evidence. No formal ROBINS-I or RoB 2 instrument was applied; risk-of-bias entries are the authors' structured qualitative judgment. Genome build and strand are not listed per row because the cited studies report variants by rsID only; readers should resolve each rsID against dbSNP (current build) before clinical interpretation.

Gene	Citation [Ref]	Variant / Effect Allele	N (analyzed)	Design	Exposure → Comparator	Primary Endpoint	Interaction p / key effect (as reported)	Prespec. / Post hoc	Multiplicity Adj.	Replication	Risk of Bias (qualitative)
APOA1	Ordovas 2002	rs670 (−75 G>A); A	1,577 (755 M, 822 W)	Cross-sectional (Framingham Offspring)	PUFA >8% E → <4% E	HDL-c	PUFA×genotype P=0.005 (women only); A-carriers +13% HDL-c at PUFA>8%, GG +14% at PUFA<4% (each P<0.05); NS in men	Observational interaction	NR	No	High (cross-sectional; surrogate; sex subgroup)
APOA1	Gomez 2010	APOA1 −76 G>A; A	97	Randomized crossover (3 diets ×4 wk)	SFA → low-fat/CHO or MUFA	LDL size; oxidation	LDL size: CHO diet ↓ size in GG; A-carriers smaller LDL + ↑ oxidation after SFA; NO oxidation interaction (exact p NR)	Prespecified genotype groups	NR	No	High (n=97; surrogate)
APOA1	Izaola 2020	rs670; A	268 (GG 179, GA 79, AA 10)	RCT, 9 mo	High-protein/low-CHO → standard hypocaloric	Lipids, HOMA-IR	A-carriers vs non: TC P=0.02; LDL-c P=0.01; insulin P=0.01; HOMA-IR P=0.03 (both diets)	Prespecified (rs670 primary)	NR	No	Moderate–high (hypocaloric confounding)
APOA1	de Luis 2019	rs670; A	282	RCT, 12 wk	High-fat → low-fat hypocaloric	Insulin resistance, HDL-c	A-carriers: insulin P=0.02; HOMA-IR P=0.01/0.03; HDL-c +4 mg/dL on low-fat P=0.03	Prespecified (rs670 primary)	NR	No	Moderate–high (hypocaloric confounding)
APOA1	Hosseini-Esfahani 2015	rs670/rs5069 + APOC3 rs5128; A/T	828 (414 case / 414 control)	Nested case-control (TLGS)	Western dietary pattern (top vs bottom quartile)	MetS	A/T carriers top WDP quartile OR 3.22 (1.21–8.58), P-int=0.03; combined APOC3/APOA1 P-int=0.003	Observational interaction	NR	No	High (composite endpoint; case-control)
APOE	Griffin 2018	ε2/ε3/ε4; ε4	389 (E4 125, E3/E3 274, E2 70)	Secondary analysis of 5-arm RCT, 24 wk (RISCK)	SFA → low-GI CHO	TC, LDL-c, ApoB	diet×genotype TC P=0.02, apoB P=0.006; E4 +low-GI CHO: TC −0.28 mmol/L (P=0.03), apoB −0.1 g/L (P=0.02)	Post hoc (retrospective genotyping)	NR	No	Moderate (surrogate; secondary analysis)

Gene	Citation [Ref]	Variant / Effect Allele	N (analyzed)	Design	Exposure → Comparator	Primary Endpoint	Interaction p / key effect (as reported)	Prespec. / Post hoc	Multiplicity Adj.	Replication	Risk of Bias (qualitative)
APOE	Carvalho-Wells 2012	ε3/ε3 vs ε3/ε4; ε4	88 (44 E3/E3, 44 E3/E4)	Sequential intervention, 8 wk/arm (SATgenε)	Low-fat → HSF → HSF+DHA	TG, CRP	genotype×diet: TG P=0.033; CRP P=0.009; no interaction for cholesterol fractions	Prespecified (prospective genotype recruitment)	NR	No	Moderate (n=88; surrogate)
APOE	Jin 2021	ε2/ε3/ε4; ε4	8,506	Prospective cohort (CLHLS)	High vs low meat / fish intake	All-cause mortality	APOE×meat & ×fish P<0.05; fish HR 0.74 (0.56–0.98) in ε4 carriers; meat HR 1.13 (1.04–1.25) in ε4 non-carriers; males only	Post hoc subgroup	NR	No (single cohort)	High (observational; confounding)
APOE	Zhang 2021	ε2/ε3/ε4; ε4	3,029	Prospective cohort (CLHLS)	Higher protein diversity / fish	Cognitive decline	Protein diversity OR 0.54 (0.34–0.88); fish OR 0.43 (0.22–0.78) in ε4 carriers	Post hoc subgroup	NR	No	High (observational; non-CV endpoint)
LIPC	Ordovas 2002	rs1800588 (−514 C>T); T	2,130 (1,020 M, 1,110 W)	Cross-sectional (Framingham)	Total fat <30% vs ≥30% E	HDL-c, subfractions	T-allele higher HDL-c only at <30% fat (P<0.001); HDL2-C P<0.001; HDL size P=0.001; SFA/MUFA not PUFA	Observational interaction	NR	No	Moderate (cross-sectional; single ancestry)
LIPC	Smith 2017	rs1800588 (−514 C>T); T	42 (+384 BPRHS subset)	Randomized crossover, 4 wk/phase	Western (high-fat) → Hispanic (low-fat)	HDL-c, TG	NS gene×diet for HDL-c (primary, null); CC/CT higher HDL-c on Western (P=0.0004/0.0003); BPRHS: SFA unfavorable in TT	Prespecified (interaction primary; null)	NR	Partial (BPRHS subset)	Moderate–high (n=42)
LPL	Hammad 2019	rs13702; CC (major)	101 (of 115 enrolled)	Randomized crossover, controlled feeding (COMIT-II)	High-MUFA canola → low-MUFA/high-SFA	Visceral / android fat	rs13702-CC: visceral fat P=0.017; android fat P=0.037 (high-MUFA vs low-MUFA)	Secondary outcome (primary null)	NR (5 SNPs)	No	High (pilot; surrogate)
LPL	Hannon 2020	rs13702; G (risk)	101	Cross-sectional (23 SNPs)	High-fat (>92 g/d) vs low-fat	HDL-c	rs13702×total fat P=0.041; G-carriers higher HDL on high-fat (56±3 vs 46±2 mg/dL, P=0.033)	Exploratory (23 SNPs)	Yes (Bonferroni)	No	Moderate–high (n=101; surrogate)

Gene	Citation [Ref]	Variant / Effect Allele	N (analyzed)	Design	Exposure → Comparator	Primary Endpoint	Interaction p / key effect (as reported)	Prespec. / Post hoc	Multiplicity Adj.	Replication	Risk of Bias (qualitative)
LPL	Ma 2014	rs320; T (major)	1,171 (+1,334 ARIC replication)	Cross-sectional (BPRHS) + replication	High vs low PUFA	BMI, waist circ.	Women: rs320×PUFA BMI P=0.002, WC P=0.001; TT higher PUFA→lower BMI (P=0.01)/WC (P=0.005); NS in minor allele	Candidate-gene; sex-stratified	NR	Yes (ARIC, AA women)	Moderate–high (sex-restricted; surrogate)
ANGPTL3	Kaviani 2019	Not genotyped (variants extrapolated)	26 (16 PUFA, 10 control)	RCT, 7-day diet (NCT02246933)	High-PUFA (21% E) → control (7% E)	Postprandial TG; ANGPTL3/8	Females only: TG AUC 141.2±18.7→80.7±6.5 mg/dL/h (P=0.01); ANGPTL3/8 ↓ in PUFA females; NS in males	Not designed for SNP interaction	NR	No	High (n=26; females; variants not measured)
FADS1/2	Hellstrand 2012	rs174547 (T>C); C	4,635 (60% F)	Cross-sectional (Malmö Diet & Cancer)	Long-chain ω-3 intake tertiles	LDL-c, HDL-c	C-allele lower LDL P=0.03; rs174547×ω-3 on LDL P-int=0.01 (effect only in lowest ω-3 tertile, P<0.001); ×ALA:LA on HDL P=0.03	Observational interaction	NR	No	Moderate (cross-sectional; European)
FADS1/2	Bäck 2022	FADS1 locus variant; minor	1,464 (transcriptomics n=410)	Cross-sectional (STANISLAS cohort)	ω-3 PUFA intake	Carotid-femoral PWV	PWV significantly associated with FADS1 variant; ω-3×genotype differential association; high ω-3 attenuated genotype effect (exact p NR in abstract)	Observational interaction	NR	No	Moderate (validated surrogate; not hard event)
FADS1/2	Manson (VITAL) 2019 / Chilton 2022	FADS not genotyped in trial (inferred)	25,871 (overall); 5,106 AA	RCT (VITAL); post-hoc race subgroup	ω-3 (EPA+DHA 1 g/d) → placebo	Myocardial infarction	Overall MACE null; African American subgroup MI HR 0.23 (0.11–0.47); race-interaction P=0.001; FADS attribution post-hoc hypothesis	Post hoc subgroup (race, not genotype)	Interaction tested (race)	No	High (FADS inferred, not measured)

Abbreviations: AA, African American; ALA, α-linolenic acid; ARIC, Atherosclerosis Risk in Communities; BPRHS, Boston Puerto Rican Health Study; CHO, carbohydrate; CLHLS, Chinese Longitudinal Healthy Longevity Study; COMIT, Canola Oil Multicenter Intervention Trial; CRP, C-reactive protein; E, dietary energy; HSF, high-saturated-fat; HOMA-IR, homeostatic model assessment of insulin resistance; LA, linoleic acid; MACE, major adverse cardiovascular events; MetS, metabolic syndrome; MI, myocardial infarction;

MUFA, monounsaturated fatty acid; NR, not reported; NS, not significant; PUFA, polyunsaturated fatty acid; PWV, pulse-wave velocity; RISCK, Reading, Imperial, Surrey, Cambridge, and Kings study; SFA, saturated fatty acid; TC, total cholesterol; TG, triglyceride; TLGS, Tehran Lipid and Glucose Study; VITAL, VITamin D and OmegA-3 TriaL; WC, waist circumference; WDP, Western dietary pattern.
